# Extradigital glomus tumor: dermoscopic description and histopathological correlation^☆^^☆☆^

**DOI:** 10.1016/j.abd.2020.11.008

**Published:** 2021-09-22

**Authors:** Lucas Campos Garcia, Ethel Nunes de Sousa Fernandes, Natália de Paiva Sobreira, Flávia Vasques Bittencourt

**Affiliations:** Hospital das Clínicas, Universidade Federal de Minas Gerais, Belo Horizonte, MG, Brazil

**Keywords:** Dermoscopy, Glomus tumor, Skin diseases, vascular

## Abstract

Glomus tumors are rare benign neoplasms arising from the neuromyoarterial structure called glomus body. They present as angiomatous papules, soft and painful, especially to cold and pressure. In general, they are solitary and affect the extremities, located mainly the subungual bed. Extradigital lesions are rare and can constitute a diagnostic challenge. This is the report of a patient with an extradigital lesion on the left arm, and its dermoscopic aspects, including angiomatous lagoons circumscribed by a pale halo, a structure not previously described in the two reports of extradigital glomus tumor with dermoscopic features, found in the literature.

## Case report

**Figure 1:**
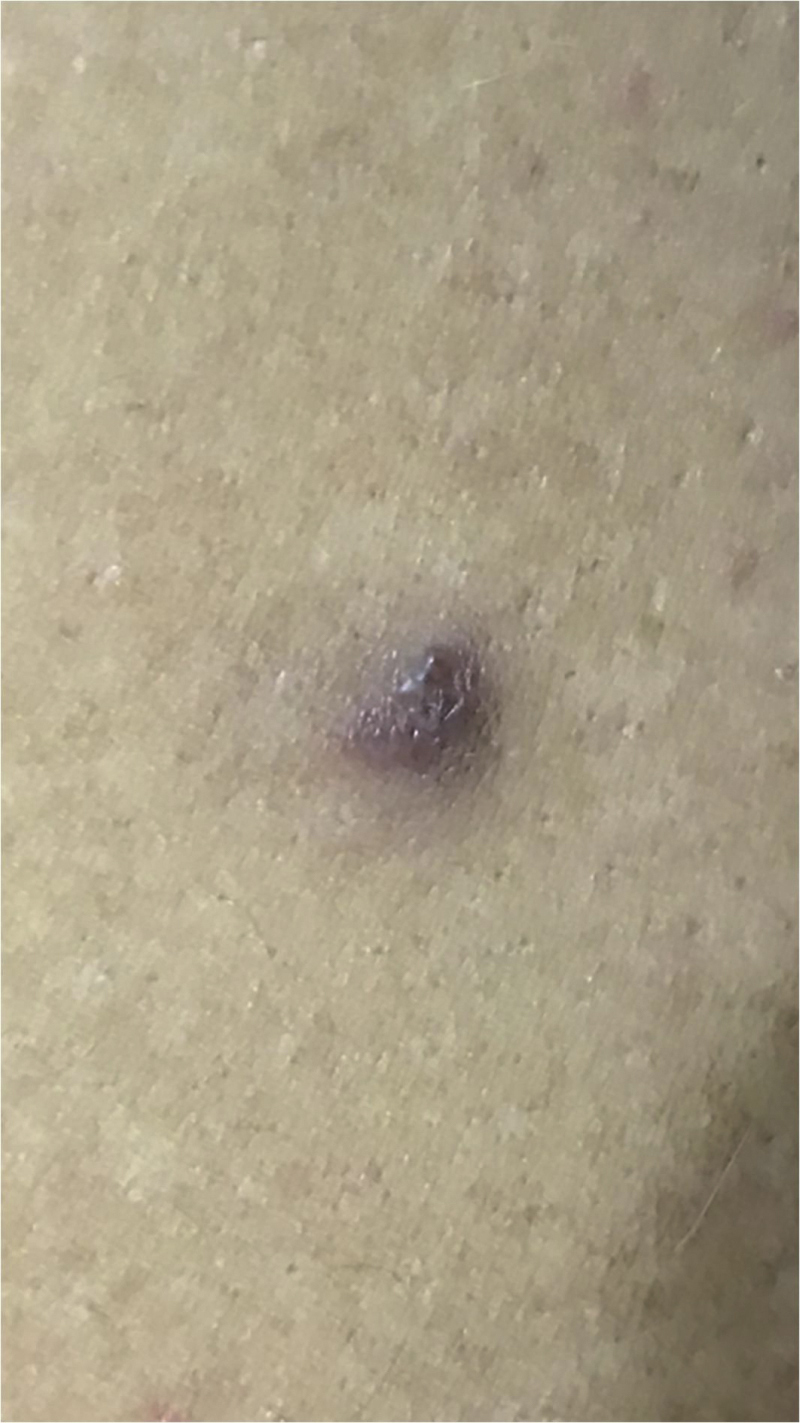
Angiomatous nodule on the lateral aspect of the left arm.

**Figure 2:**
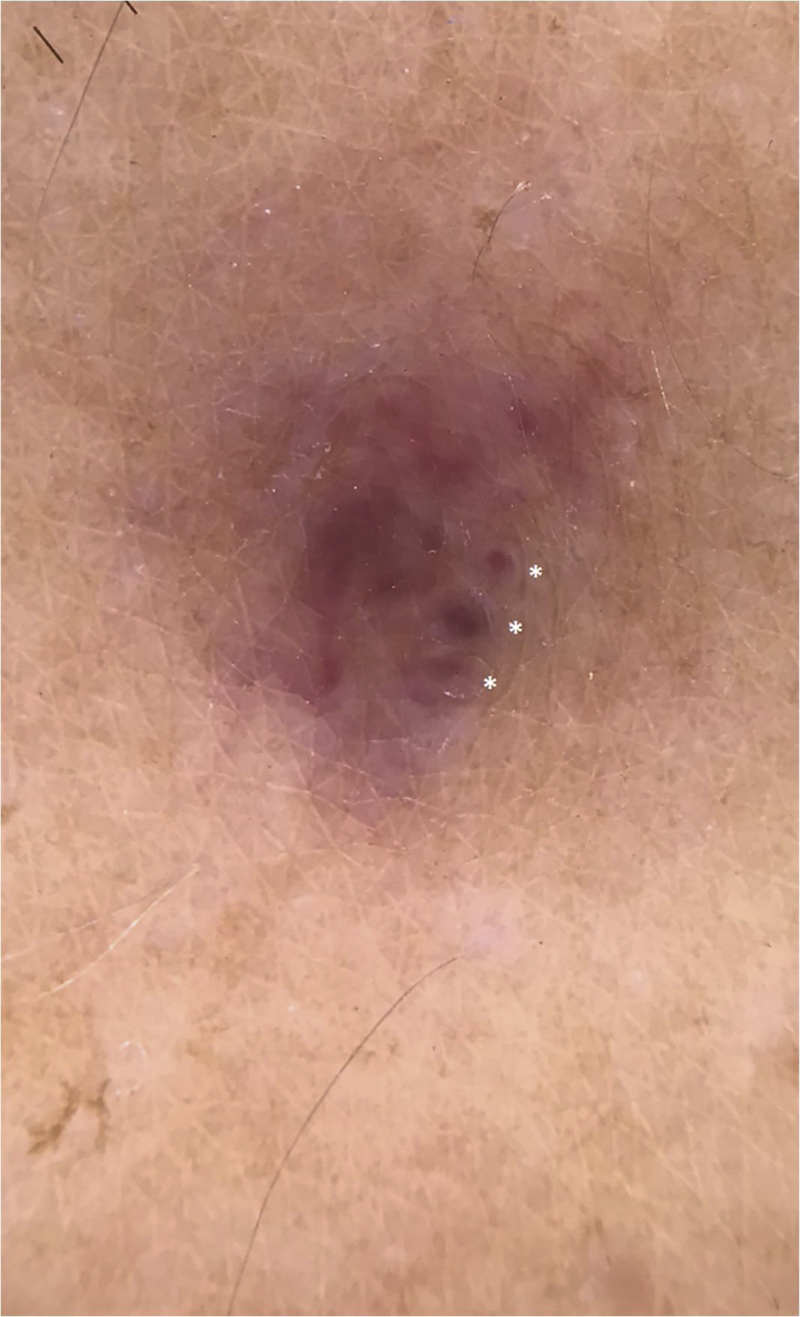
Dermoscopy showed a homogeneous purplish area without structures surrounded by a whitish region, in addition to intensely purplish lagoons, standing out individually and surrounded by a pale halo (asterisks).

**Figure 3:**
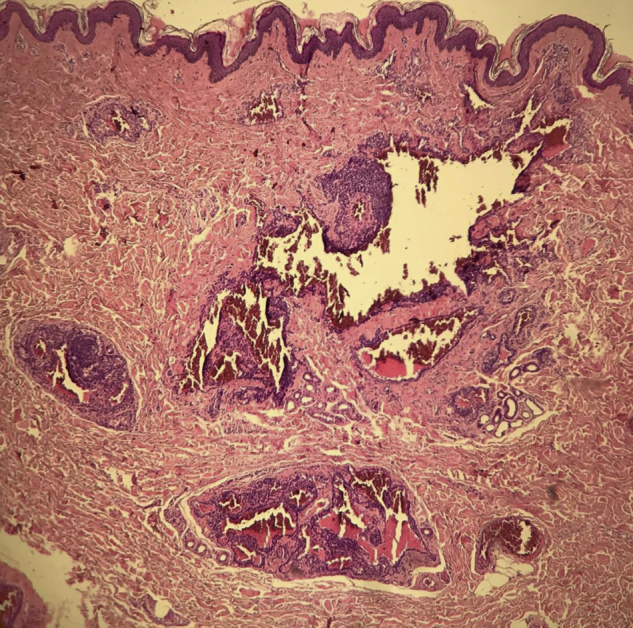
Histopathology showed intradermal nests of monomorphic glomus cells, with rounded nuclei, organized in single or multiple cords around exuberant vascular structures, corresponding to a glomangioma. (Hematoxylin & eosin, ×40).

**Figure 4:**
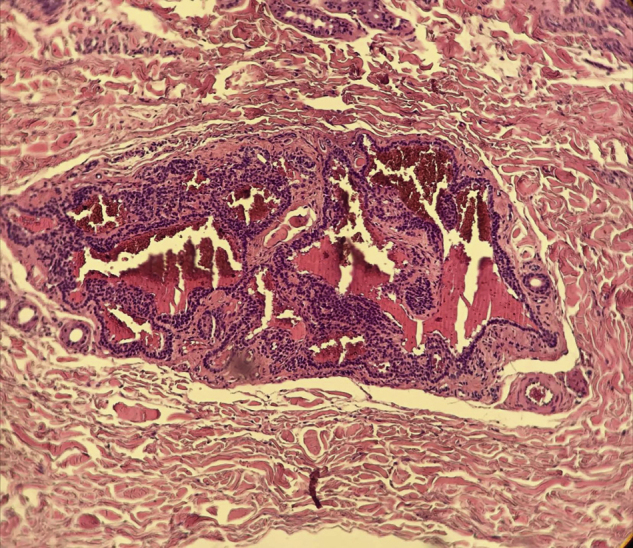
At the center of the histological section, there is a nest consisting of glomus cells, with regular and monomorphic nuclei, surrounding vascular spaces. The adjacent dermis shows thickened collagen fibers. (Hematoxylin & eosin, ×400).

A 35-year-old male patient complained of a painful angiomatous nodule on the lateral aspect of the left arm for about eight years (Fig. 1). He reported worsening of the pain with exposure to cold and digital pressure. Love’s test was positive and Hildreth’s test was not performed due to the anatomical difficulty in properly positioning the tourniquet. Dermoscopy revealed purplish lagoons circumscribed by a pale halo next to a pink area without structures (Fig. 2). Surgical excision of the lesion was performed and histopathological confirmation of extradigital glomus tumor (EGT) was attained. The histopathological analysis showed intradermal nests of monomorphic cells, with rounded nuclei, organized in single or multiple cords around vascular structures (Fig. 3). In the center of the histological section, one of these nests can be seen, showing exuberant size in relation to the others (Fig. 4). There has been no recurrence after two years of follow-up.

## Discussion

Glomus tumors (GTs) are rare benign neoplasms of the normal neuromyoarterial glomus body, constituting 1.6% of all soft tissue tumors.^1^, ^2^ GTs tend to be solitary, located in the digital region (usually subungual), of dark blue to purple color, and accompanied by the classic triad of spontaneous pain, hyperalgesia to digital pressure, and sensitivity to cold.^1^, ^2^, ^3^, ^4^

EGTs correspond to approximately 26.7% of GT cases, with a higher incidence in middle-aged (mean age 48) and male patients (11:3). The upper limbs are the most frequently affected site.^1^ In these cases, the symptomatic triad may be incomplete.^1^, ^5^ Due to the clinical manifestations and unusual locations, only 20% of cases receive the correct initial diagnosis.^1^, ^2^

The differential diagnoses include other painful nodular tumors, especially eccrine spiroadenoma, leiomyoma, neurofibroma, and dermatofibroma. In addition to these, clinically and dermoscopically, they must be differentiated from angiomatous lesions, such as angiomas and angiokeratomas. Imaging tests, such as ultrasound and nuclear magnetic resonance can help to attain the clinical diagnosis.^6^ The present patient had the complete triad and additional imaging exams were not necessary.

Dermoscopy is a non-invasive method that can help manage these cases.^2^, ^3^ However, even when considering the most prevalent clinical form, digital GTs, there are few studies available in the literature regarding the dermoscopic features.^7^ After an extensive literature search, the authors found only two dermoscopic descriptions of EGT.^4^, ^8^ In both, a homogeneous, unstructured purplish area surrounded by a whitish region is reported.^1^, ^4^ However, in the present case, in addition to the aforementioned classical findings, intensely purplish lagoons were also demonstrated, standing out individually from the pinkish area without structures and circumscribed by a pale halo (Fig. 2). After reviewing the literature, no previous description of this finding was found.

On histopathology, GTs have varying proportions of glomus cells, blood vessels, and smooth muscle. They are classified according to these characteristics into the following subtypes: solid glomus tumor (SGT), glomangioma, or glomangiomyoma.^1^, ^6^, ^9^

SGT consists of nests of uniform grouped glomus cells, without prominent vascularization or exuberant smooth muscle tissue. Tumors with differentiation and the significant presence of smooth muscle tissue are subclassified as glomangiomyomas. Glomangiomas show intradermal nests of monomorphic glomus cells, with rounded nuclei, organized as single or multiple cords, around exuberant vascular structures.^1^, ^2^, ^4^, ^6^ The description is consistent with that of the present case (Fig. 3). The prevalence of glomangiomas is highest among extradigital tumors.^1^

The dermoscopic finding of a purplish area without structures, previously described in the literature, was found in SGT lesions constituted by a cohesive tumor with a more abundant cell component in relation to the vascular spaces. This histopathological organization is reflected in the observed dermoscopic findings.^4^ In glomangiomas, the large vascular structures surrounded by delicate cell cords may stand apart and stand out individually (Fig. 4), originating the purplish lagoons described in this study. Thus, the authors believe that it is not only a dermoscopic finding associated with EGT, but it also indicates a specific subtype: glomangioma. Despite the limitation of conclusions from a single case, this new dermoscopic finding can complement the investigation and increase the accuracy of the diagnosis of EGT.

## Financial support

None declared.

## Authors’ contributions

Lucas Campos Garcia: Approval of the final version of the manuscript; drafting and editing of the manuscript; critical review of the literature; critical review of the manuscript.

Ethel Nunes de Sousa Fernandes: Approval of the final version of the manuscript; drafting and editing of the manuscript; critical review of the literature.

Natália de Paiva Sobreira: Approval of the final version of the manuscript; drafting and editing of the manuscript; critical review of the literature.

Flávia Vasques Bittencourt: Approval of the final version of the manuscript; critical review of the literature; critical review of the manuscript.

## Conflicts of interest

None declared.
